# Cultural Beliefs and Traditional Practices During Pregnancy, Child Birth, and the Postpartum Period in East Gojjam Zone, Northwest Ethiopia: A Qualitative Study

**DOI:** 10.1089/whr.2023.0024

**Published:** 2023-08-16

**Authors:** Bewket Yeserah Aynalem, Misganaw Fikrie Melesse, Yibelu Bazezew Bitewa

**Affiliations:** Department of Midwifery, Debre Markos University, Debre Markos, Ethiopia.

**Keywords:** East Gojjam, cultural beliefs, traditional rituals, childbirth practices, Ethiopia

## Abstract

**Introduction:**

Many countries invest in interventions to minimize maternal and neonatal morbidity and mortality but the progress has been slow, in developing countries, especially in Africa. Traditional taboos and malpractices like home deliveries owing to cultural beliefs and traditional practices during pregnancy, childbirth, and the postpartum period increase maternal and neonatal complications. Although there are different researches in Ethiopia, the reasons for practicing such traditional activities in the East Gojjam zone in northwest Ethiopia are not well understood. Ethical clearance was obtained from the research committee of Debre Markos University.

**Objective:**

This study aimed to explore cultural beliefs and traditional practices during pregnancy, childbirth, and the postpartum period in East Gojjam Zone, Northwest Ethiopia.

**Materials and Methods:**

Purposive and snowball nonprobability sampling techniques were used to select the study participants. Data were collected through in-depth interviews and focused group discussions until the information was saturated and it was categorized and analyzed after the interviews were completed. The translated text file was analyzed using thematic analysis using codes and terms to create themes. Information from the interview consists of the women's descriptions and explanations of their cultural practices during pregnancy, childbirth, and the postnatal period.

**Result:**

Respondents report many examples of cultural, traditional, and religious practices experienced by the community in East Gojjam Zone during pregnancy, childbirth, and postpartum period, whether they are beneficial or harmful for the mother and the fetus. Drinking holy water, praying to God and Mary, taking herbal medicine, food taboo, making a confinement period in a dark room, and other cultural ceremonies are among commonly experienced cultural, traditional, and religious practices in the study area.

**Conclusion:**

Traditional, cultural, and religious practices during pregnancy, childbirth, and the postpartum period are still popular among the community in the East Gojjam Zone. It is critical to identify the harmful practices and reinforce the positive healthy practices to make pregnancy, childbirth, and postpartum periods healthy and joyful.

## Introduction

Culture is a socially shared view and behavior practiced in a certain society.^1,2^ Some beliefs, customs, and rituals can be advantageous, and others are harmful or have no impact on the health of the mother or her child in varying levels and types from area to area.^3^ Although childbearing is a universal experience, multiple cultures surrounded maternal and child health, some can be advantageous, and others are harmful or have no impact on the health of the mother or her child in varying levels and types from area to area.^1,4^ Cultural beliefs and attitudes may influence health-seeking behaviors, which in turn contribute to a compromised state of maternal and child health. There are some controversies on the importance of cultural knowledge and beliefs.^5–7^ Traditional practice is one of the factors that have a great impact on health and health care utilization. The maternal and child health care service utilization especially in the rural part of the zone is very poor and not standardized.^8,9^

Despite good progress in reducing infant and under-5 mortality in Ethiopia, maternal and neonatal mortality is not getting reduced as expected.^10,11^ The current fertility rate in Ethiopia is 4.24 births per woman and in remote areas; the birth interval is too short, which in turn affects the whole life of the women, children, and the family. The fertility rate of women at a young age in rural parts of the country increased as compared with women at a young age in urban areas.^12^ Maternal and child health are a valuable component of public health improvement in the world.^13,14^ Safe Motherhood programs were started worldwide to improve maternal and perinatal health. Insufficient maternity services are one of the main contributing factors to maternal and perinatal morbidity and mortality.^15^ There is lack of appropriate health services like shortage of both human and financial power; low access to maternal and perinatal health services; and lack of knowledge in reproductive health and related issues like poor awareness of safe motherhood and contraceptive services.^16,17^

The Ethiopian-2019 demographic health survey showed that there was a low coverage of antenatal care (ANC); 43% of women had at least four ANC visits during their last pregnancy. Institutional deliveries increased from 26% in 2016 to 48% in 2019, whereas home deliveries decreased from 73% to 51% over the same period; 34% of women and 35% of newborns received a postnatal check within the first 2 days after birth.^12^ Residence was one factor that influences institutional deliveries owing to cultural practices.^18,19^ Feeling no need to go to a health institution, residence (distance), and some health costs were the main contributing factors to home birth. A majority of births were also assisted by family and relatives and some are assisted by traditional birth attendants (TBAs).^20–22^ More urban mothers could get senior birth attendants as compared with those mothers who give birth at remote health institutions.^23^

Socioeconomic and cultural-related reasons are the contributing factors impeding mothers to get services in health institutions.^24,25^ Laboring mothers who have more supporting people during the perinatal period will have a chance to get fewer complications.^26,27^ In Ethiopia, health extension workers (HEWs) are supported by women in the Health Development Army who meet regularly to discuss issues related to women's and children's health. This includes topics such as vaccination, sanitation, and reducing harmful traditional cultural practices during pregnancy, childbirth and the postpartum period, and so on.^28^

The delay in decision-making, delay in getting transport, and delay in getting treatment/services still contribute to maternal and perinatal complications.^29,30^ Identifying the underlying reasons for maternal mortality like home delivery, attended by unskilled birth attendants, and other reasons are important to explore the belief, attitudes, and practices that make gravid mothers decide that it is not important to go to health facilities like health centers and hospitals for ANC, childbirth, and postpartum care services.^31,32^ The traditional, cultural, and social aspects of Ethiopian mothers from urban and rural areas especially in the East Gojjam zone are still not explored and identified. As shown in a previous study, the magnitude of home delivery and traditional and cultural practices in Ethiopia especially in the East Gojjam zone is huge.^9^

Thus this study aimed to explore cultural beliefs and traditional rituals about childbirth practices in East Gojjam Zone, Northwest Ethiopia, which help to have clear information on these traditional and cultural practices that may be included in the health intervention programs to minimize the maternal and neonatal mortality and morbidity in Ethiopia, especially in the study area.

## Materials and Methods

### Study area and period

East Gojjam zone is located in the Amhara region 300 km from Addis Ababa, the capital city of Ethiopia, and 265 km from Bihar Dar, the capital city of Amhara. It is bordered on the south by the Oromia Region, on the west by West Gojjam, on the north by South Gondar, and the east by South Wollo. East Gojjam zone is one of the poor zones in the Amhara region, Northwest Ethiopia, and as the zone health office report showed East Gojjam zone has a total population of 2,719,118 and 632,353 households. East Gojjam zone has also 21 Woreda, 480 Kebeles, 423 health posts, 102 health centers, 10 primary hospitals, and 1 comprehensive specialized hospital. This study was conducted in the East Gojjam Zone, Amhara region, Northwest Ethiopia from January 2022 to May 2022.

### Participants

Qualitative research methods were used in this study including focus group discussions (FGDs) and in-depth interviews (IDIs). Those mothers who had a history of home delivery in the last year, their families, community leaders, and TBAs were the key informants. Women who gave birth in the past year with exceptional pregnancy outcomes specifically those who had obstructed labor, whose family had complications or expired, and whose family had an invaluable role in deciding on pregnancy/labor delivery/postnatal period were included in the study.

### Data collection

Purposive sampling techniques were used to select the respondents from the study area, after a detailed discussion with the selected health offices, health institutions, and community leaders. The respondents were selected through the HEWs and community leaders. The Snowball sampling technique was also used to select the respondents by getting their friends or families, whatever the birth outcome either bad or good, and those who were interested to be included in the study. Similarly, HEWs and TBAs also participated in the study. Each individual in the study was briefed about the study by the principal investigators, then after obtaining informed consent and place of the interview was arranged between the principal investigators and the interviewees.

The questions progressed conversationally and forwarded from general to specific and from easy to difficult. Because the questions were open-ended and the respondents were allowed to do most of the talking, the wording and sequence of questions were adjusted to meet the local culture and feelings of the respondents. All interviews were conducted at the date and times settled by the respondents and in places where the respondents feel at ease talking. Interviews were held in a private setting by the principal investigators. The interview was conducted in the morning and lasted 30–90 minutes.

### Data analysis

Qualitative data were analyzed using content analysis. Verbatim transcription in the Amharic language was made to convert the raw notes and audio files into a text file. The transcribed text file was translated into the English language for analysis. The investigators read the transcripts many times to gain a better understanding of the context and then coded, identifying categories and major themes. The translated text file was analyzed using thematic content analysis using codes and terms to create themes. Information from the interview consists of the women's descriptions and explanations of their cultural pregnancy and childbirth practices.

### Ethical considerations

Ethical clearance was obtained from the research committee of Debre Markos University and was submitted to the East Gojjam zone health bureau. A letter was also obtained from this Bureau and the investigators received a permission letter before directly contacting other concerned bodies and the study participants. The objective of the study was explained, and written informed consent was obtained from each participant before the interview and discussion. The interview was conducted in private places to maintain the privacy of study participants. Participant records and identifiers were kept anonymous by giving codes instead of personal identifiers such as names. Participants who were unwilling to participate and wanted to withdraw at any step of the interview had the freedom to do so without any restriction.

## Result

### Sociodemographic characteristics

Seventy-two (8 FGDs every 6 discussants and 24 IDIs) study participants were included in the study. Among the total study participants, four FGDs were men and all the participants in the IDIs were women. The age of the respondents was between 18 and 41 years for IDI, whereas the age of FGD respondents was from 21 to 46 years. Nine and 15 women from IDIs and FGDs were unable to read and write, respectively. One case from IDI and one case from FGD had a history of abortion and early neonatal loss, respectively. Four study participants from IDI and six discussants from FGD had a history of home delivery. Two (8.3%) from IDI and 4 (16.7%) female discussants developed obstetrics complications.

Almost all the respondents were Orthodox Christians and only three of them were Muslims. The majority of household heads were men (22 men and only 2 women). Their age ranged from 31 to 68 years. All of them were educated either formally or informally like spiritual or adolescent education.

### Cultural practices and beliefs during pregnancy

In East Gojjam Zone, many cultural practices and beliefs are experienced during the antenatal period for different reasons.

“I drink a potent tella after 8 months of pregnancy because when I take it, my fetus moves actively that makes my lung free and I feel a rest.” (A 32 years old, female discussant)

The majority of husbands in the study area are actively involved in giving care to their wives during pregnancy according to their culture and traditional beliefs because husbands are the head of the household and the decision-maker about the health of the families.

“My wife should be free from any activity and restricted from a long journey when she became pregnant because I believe that strenuous activities may lead to abortion.” (Husband, 37 years old)

Although a more balanced diet is recommended during pregnancy by WHO, culturally, many women restrict themselves from taking nutritious foods like butter, banana, milk, honey, and meat for different cultural beliefs. A woman who was involved in IDI said that “I never take banana and honey while I was pregnant because these foods may stick on my fetus's body and head that make a scar and hairless.” (39-year-old woman)

Many study participants do not take these foods during pregnancy for fear of macrosomia that prolongs labor and other complications. “Most pregnant women prohibited from eating butter and meat throughout their pregnancy period because these foods may prosper the fetus that will result in a bad outcome during labor.”(A 55-year-old community leader)

On the contrary, some other study participants believe that taking such nutritious foods leads pregnant women to the fat that makes their birth canal narrowed and unable to deliver easily. “I believe that these delicious foods may make my body fatty including my pelvic wall during pregnancy period that makes it very difficult for the baby to pass through it. Due to this, I don't take such foods while I became pregnant.” (A 34-year-old woman, involved in IDI)

On the contrary, communities in the study area strongly recommend that some traditional foods and herbs should be taken by pregnant women to prevent Rh-isoimmunization locally known as “shotelay” and other pregnancy complications. “I always take an herbal medicine at 7 months of my pregnancy to prevent fetal death due to shotelay.” (A 41-year-old woman, involved in IDI)

The other community leader discussant said that “I always feed my wife barley bread, grinded pepper, and roasted grain during her pregnancy because these food items are light for her and the fetus.” (Community leader, 64 years old)

Most of the discussants agreed that drinking local herbal drinks like damakasiye, grawa, aregresa, tenaadam, rue, and garlic had a positive effect on pregnancy outcomes.

The rural community in the study area is highly dependent on religious rituals in their daily life particularly when someone becomes unhealthy or ill. They consult religious leaders, drink holy water, and pray to God in the Church in front of the Saint's picture. They believe pregnancy by itself is becoming unhealthy. “Pregnant mothers always drink holy water at which Saint Rufael's missal prayed throughout their pregnancy period because they believed that Saint Rufael is authorized by God to help women in labor for safe delivery without any complication.” (HEW, 31 years old)

The other discussant said that “My family obligates me to wear a magical paper on my neck while I became pregnant because this magical paper saves me from Satan that aborts, convulses, bleeds and kills my offspring and me during my pregnancy period.” (A 34-year-old female discussant)

### Cultural practices and beliefs during childbirth

Childbirth is the normal physiology of human beings at which two extreme events occurred. The most severe pain in the life of women exists during labor and delivery to end up with a joyful coming of a newborn that eases their labor pain. In Ethiopia, this event is surrounded by many cultural practices, traditional beliefs, and rituals to facilitate the delivery process at home. About 32.3% of women still delivered at home in East Gojjam Zone for different cultural reasons.^33^ One woman explained that during prolonged labor she would sit on the hyena's leather when the labor is prolonged, which fastens her delivery. (Female discussant, 43 years old)

There are other cultural beliefs and traditional rituals practiced during childbirth in the East Gojjam Zone—like polishing the house with cow dung, opening every object in the house, losing the husband's belt, kneeling on muck, reflecting the mirror, crossing the laboring woman across the river, sitting on the place of dough container, roasting barley three times, making coffee repeatedly, moving a white chicken around the laboring mother's head three times, massaging the laboring mother's abdomen with butter and others.

“…..In my community, husbands open all the ceilings of the house when their wives' labor is prolonged to visualize the sky and to have a direct communication with Almighty God …….” (Community leader, 67 years old)

The other study participant involved in the IDI said that “….I prepare a white chicken when my delivery date is reached and if my labor is prolonged, the TBA will move the chicken around my head three times to drive away the Satan from my head that prolongs my labor” (Woman, 35 years old)

A female participant from FGD said that “When my labor is prolonged, my families think as the Satan is the cause and they carry and take me across a big river to make the Satan leave me and enter the river so that I will deliver immediately…” (Woman, 21 years old)

A TBA who was involved in the IDI said that “……when a laboring mother's pain becomes severe, I will massage her abdomen with butter in a downward fashion to shorten the labor…..” (TBA, 46 years old)

Traditional rituals and spiritual beliefs are also practiced in rural areas of East Gojjam Zone during labor and delivery at which home delivery is common.

“…..All family members and neighbors will carry a hot stove and pray towards the Almighty God by saying mercerize! Lord, mercerize…us, Lord mercerizes…us Lord…locally known as Egzio meharene Christos! Egzio meharene Christos! Egzio meharene Christos…” (A priest husband, 49 years old)

A woman involved in the IDI said that “…..when the labor becomes tense and prolonged, all people in the house will pray to Mary by saying Mariam! Mariam! Mariam! By putting Mary's picture on laboring mother's abdomen….” (Woman, 45 years old)

### Cultural practices and beliefs during the postpartum period

The postpartum period is a time of transition and social celebration in many societies, signaling an adjustment of cultural responsibilities. Traditionally, a woman remains at home, and leaving the mother alone is forbidden during this period. During this time, her behavior with diet, activity, and hygiene is determined by tradition, culture, and rituals.

### For placenta delivery

Culturally there are lots of activities practiced for safe delivery of the placenta during home delivery by the community in the study area. The majority of the discussant agreed that both the mother and the newborn had never taken anything before the delivery of the placenta.

“…When the placenta cannot be delivered easily I insert butter-smeared spindle to the mother's throat to expel the placenta while the mother sneezes and coughs…..” (TBA, 44 years old)

The other male discussant said that … “When the placenta will not be delivered immediately, we tie the cord by the thread with a pestle and pull it till it will be expelled…” (Husband, 58 years old)

A woman involved in the IDI said that “when I was sat at the dry cow dung, the placenta was delivered immediately….” (Woman, 26 years old)

### To control massive bleeding after delivery

Bleeding after childbirth is fatal for a woman especially delivered at home because no uterotonic drug is given. However, there are a lot of traditional and cultural measures taken by the community to control postpartum hemorrhage.

“Shooting the gun and knocking the griddle (creating a harsh sound) nearby the delivered mother effectively stops bleeding because when the mother accidentally heard the sound of a gun, she shivers and her blood will be distributed to her head and other body parts rather than bleeding out….” (Husband, 63 years old)

The other male discussant said that “when there is massive bleeding after delivery, we will take the woman to the mountain to stop bleeding by being far from evil spirit living at home that facilitates bleeding for his drink” (Husband, 53 years old)

A HEW said that “when there is active bleeding after delivery, the woman holds the silver cross with her teeth and stays in lying head down position” (HEW, 27 years old)

### Cultural practices when the postpartum period is safe

This is the period of happiness for the mother, the family, and neighbors in general and they celebrate together with different cultural ceremonies including preparing porridge, slaughtering a sheep, goat, or hen, making coffee, and others. Praying to Mariam is acceptable in Orthodox Christians in Ethiopia,^34^ and this activity has also been practiced in the east Gojjam zone.

Most of the discussants said that …. “The first thing that should be done immediately after safe delivery is preparing porridge to see Saint Mary off ….”

A woman involved in the FGD said that “I live in a dark room for a month with a charcoal fire to make a room warm.” (Woman, 29 years old)

The other discussant said that “I never drink water for 20 days after delivery to prevent the neonate from abdominal cramp.” (Woman, 33 years old)

A woman involved in the IDI said that “My husband breaks a salt bar on my bed by the 3rd day of delivery.” (Woman, 40 years old)

In the East Gojjam zone's culture, all Orthodox Christian followers take a holy shower on the 20th day of delivery by the religious leader. After taking a holy shower, the woman can touch an object and get out of her bed for having sunlight.

A woman involved in IDI said that “I wrap malt with a leaf and put it on the neonate's head to avoid tonsillitis.” (Woman, 28 years old)

## Discussion

This study explored and describes the cultural and traditional beliefs about pregnancy, childbirth, and postpartum practices in the East Gojjam Zone. The findings revealed that most participants practiced traditional childbirth rituals and practices during pregnancy, delivery, and postpartum periods. Our research supports the view that cultural rituals are important in pregnancy, childbirth, and puerperium.^1,5,6^

The traditional, spiritual, and cultural beliefs as well as decision-making power within the household influence the childbirth practice and choice of the place of delivery. In East Gojjam Zone, the cultural and traditional beliefs and rituals related to pregnancy and childbirth are aimed to preserve the life and well-being of the mother and her baby. This is similar to the biomedical mode but differs in terms of the immediate social context in which they act, and of the cultural values that they practiced.^2^ This finding showed that foods like bananas, meat, and milk products were prohibited during pregnancy. This result is supported by other studies carried out in Ethiopia^33,35,36^ and Ghana.^37,38^ The possible explanation may be owing to their similarity in socioeconomic status, civilization, and cultural sensitivity. On the contrary, some local drinks like “tella” and foods like roasted grain were permitted to be taken by pregnant mothers.

In contrast to this physical activity, restriction is common in the study area during pregnancy. These findings were supported by a study carried out in Turkey.^39^ Taking herbal medicines during pregnancy is the other finding of this study for different purposes, which are supported by other studies carried out in the Western Region of Ghana^37^ and Nepal.^40^ Abdominal massage during labor to facilitate the birth of the fetus is another cultural practice in the study area, which is supported by the studies performed in Ethiopia.^33,41,42^ Praying to God is a common religious practice when labor is prolonged in the study area, which is similar to the other study conducted in Ghana.^37^ The finding of this study also showed that shooting the gun and knocking the griddle are among the cultural measures taken by the community to manage postpartum hemorrhage in the East Gojjam zone.

Living in a dark room by taking porridge as the main food with water restriction for 20 days is the other cultural practice in the study area. This finding is supported by a study carried out in South India,^43^ but in contrast with the study conducted in Malaysia.^44^ The health development army and HEWs can create awareness about the disadvantages of cultural practices and danger signs during pregnancy, labor, and the postpartum period, which will assist those to seek health care services timely; assist and refer the laboring mother to health institutions. This finding was supported by the study carried out in Ethiopia.^45^

## Conclusion

Traditional, cultural, and religious practices during pregnancy, childbirth, and the postpartum period are still popular among the community in East Gojjam Zone. It is critical to identify the harmful practices and reinforce the positive healthy practices to make pregnancy, childbirth, and postpartum period a healthy and joyful period for the mother and the newborn. Drinking holy water, praying to God and Mary, taking herbal medicine, food taboo, making a confinement period in a dark room and other cultural ceremonies are some cultural and traditional practices during pregnancy, labor, and the postpartum period in the east Gojjam zone, Northwest Ethiopia. Further research is advisable on which cultural practices and traditional beliefs are important and harmful during pregnancy, childbirth, and after delivery.

## Data Availability

The data contain personal and culturally sensitive information that cannot be shared.

## Abbreviations Used

ANC: antenatal care
FGD: focus group discussion
HEW: health extension worker
IDI: in-depth interview
TBA: traditional birth attendant
